# Artificial intelligence in eating disorder research: a scoping review with bibliometric analysis of trends, methodologies, and translational readiness (2016–2025)

**DOI:** 10.1007/s40519-026-01905-w

**Published:** 2026-09-20

**Authors:** Yasin Caliskan, Mesut Sari, Hülya Binokay

**Affiliations:** 1 Department of Child and Adolescent Psychiatry, Istanbul Basaksehir Cam and Sakura City Hospital, University of Health Sciences, G-434 Street No: 2L Basaksehir, Istanbul, Turkey; 2 Department of Child and Adolescent Psychiatry, Bolu Abant Izzet Baysal University, Faculty of Medicine, Bolu, Turkey; 3 Department of Biostatistics, Çukurova University School of Medicine, Adana, Turkey

**Keywords:** Artificial intelligence, Machine learning, Eating disorders, Bibliometric analysis, Translational research

## Abstract

**Purpose:**

Artificial intelligence (AI) applications in eating disorder research have expanded rapidly, yet the structure, scope, and translational readiness of this literature have not been systematically mapped. This study aimed to characterise publication trends, AI methodologies, clinical application domains, data sources, and implementation readiness across the empirical literature on AI in eating disorders.

**Methods:**

A scoping review with bibliometric analysis was conducted across three databases (PubMed [MEDLINE], the Web of Science Core Collection, and Scopus) on 20 November 2025. Studies were included if they reported original empirical research employing AI or machine learning as the primary analytical method in an eating disorder population. A total of 66 articles published between 2016 and 2025 were included. Each study was classified according to predefined taxonomies covering AI methodology, clinical application domain, data source type, implementation readiness, eating disorder type, and target population. Bibliometric indicators, including total citations, citations per year, and normalised citation counts, were retrieved from the Web of Science Core Collection. Co-authorship, keyword co-occurrence, bibliographic coupling, and co-citation networks were constructed using Python (version 3.11) with the NetworkX and matplotlib libraries, and reporting quality was appraised using the TRIPOD+AI and CONSORT-AI statements.

**Results:**

Publication output grew from one article in 2016 to 16 in 2025. Machine learning was the dominant AI methodology (78.8%), followed by natural language processing (19.7%) and a single conversational AI application (1.5%). The most common clinical applications were risk prediction (33.3%), diagnosis (25.8%), and treatment outcome prediction (21.2%). Clinical data (42.4%) and neuroimaging (18.2%) were the most frequently used data sources. The majority of studies remained at the laboratory stage (77.3%), with only two describing clinically deployed systems. The United States contributed 33.3% of articles; no contributions were identified from Africa, South America, or Southeast Asia.

**Conclusions:**

AI research in eating disorders is growing rapidly but remains geographically concentrated, methodologically dominated by traditional machine learning, and largely confined to laboratory settings. Adolescent populations (despite representing the peak age of eating disorder onset), bulimia nervosa, and low- and middle-income country contexts are systematically underrepresented. Closing the gap between research output and clinical implementation will require independent model validation, prospective evaluation, and sustained multidisciplinary collaboration.

*Level of Evidence* Not applicable. This is a scoping review with bibliometric analysis that maps the scope and structure of a research field rather than evaluating clinical effectiveness; it therefore does not correspond to a traditional level-of-evidence rating.

**Supplementary Information:**

The online version contains supplementary material available at https://doi.org/10.1007/s40519-026-01905-w.

## Introduction

Eating disorders (EDs) represent one of the most challenging psychiatric conditions, characterised by high chronicity, treatment resistance, and the highest mortality rate amongst all mental health disorders [1–3]. Anorexia nervosa (AN), bulimia nervosa (BN), and binge eating disorder (BED) affect millions worldwide [4], yet more than half of patients fail to achieve full recovery even with evidence-based treatment [5]. The complexity of these disorders stems from their multifactorial aetiology—involving biological vulnerabilities, psychological traits, and sociocultural factors—and is compounded by high rates of psychiatric and medical comorbidities [6, 7]. Traditional diagnostic approaches rely heavily on clinical interviews and subjective assessments, which are time-intensive, prone to bias, and limited in their capacity to identify subtle patterns or predict treatment outcomes [8]. Moreover, access to specialised care remains severely constrained by workforce shortages and long waiting lists [9], creating an urgent need for innovative, scalable solutions to improve both diagnostic accuracy and therapeutic effectiveness.

Artificial intelligence (AI) and machine learning (ML) technologies have emerged as a transformative frontier in mental health care, offering unprecedented opportunities to analyse complex, multimodal datasets and identify patterns that may elude human observation [10, 11]. In the context of eating disorders, AI-driven approaches span a wide range of applications: from developing objective diagnostic tools based on neuroimaging biomarkers [12, 13] and genomic data [14, 15], to predicting treatment response through analysis of clinical, behavioural, and physiological indicators [16, 17]. Natural language processing (NLP) techniques have enabled the detection of early warning signs in social-media content [18, 19], whilst wearable devices integrated with machine learning algorithms facilitate real-time symptom monitoring and intervention delivery [20]. Additionally, AI-powered administrative tools—such as automated clinical documentation—have been explored as a means to reduce clinician burden, though current evidence highlights significant challenges in accuracy and reliability that must be addressed before widespread adoption [21]. Recent surveys indicate that 59% of clinicians working with eating disorders have already begun incorporating AI tools into their practice, with ChatGPT being the most frequently used application [9]. This rapid adoption reflects both the potential of AI to address critical gaps in care delivery and the growing recognition that computational approaches may complement traditional clinical expertise in meaningful ways.

Despite this promise, the integration of AI into eating disorder care is not without significant risks and challenges. The case of the Tessa chatbot serves as a cautionary example: initially developed as a rule-based intervention and validated through rigorous randomised controlled trials [22], the chatbot was later implemented with an improperly enabled generative AI feature that began providing harmful dieting advice and encouraging weight loss, ultimately leading to its suspension [23, 24]. This incident illustrates that risks lie not only in the technology itself but also in implementation processes, oversight mechanisms, and the potential for misalignment between AI outputs and clinical best practices [25]. Beyond safety concerns, ethical challenges abound, including questions of algorithmic bias [26, 27], patient autonomy, data privacy, and accountability when AI tools contribute to clinical decision-making. Legal and regulatory frameworks have struggled to keep pace with technological innovation, leaving ambiguity around liability and standards of care [28]. Furthermore, the rapid evolution of AI methods often outpaces the slower, more rigorous validation processes required in clinical research, creating a translational gap between laboratory development and real-world implementation [29]. As the field moves forward, the critical question is not whether to adopt AI, but how to integrate it responsibly, ethically, and effectively.

Despite the growing body of research at the intersection of AI and eating disorders, the field remains fragmented across diverse disciplines, methodologies, and clinical applications. To date, no comprehensive synthesis has mapped the intellectual landscape of this emerging area, leaving critical questions unanswered: Which AI techniques and algorithms are most frequently employed? How is research distributed across different eating disorder types and target populations? What clinical applications—from diagnosis and risk assessment to treatment prediction and monitoring—are being prioritised? Where are the geographical concentrations of research activity, and which regions remain underrepresented? Most importantly, what is the magnitude of the gap between laboratory-based AI research and clinically validated, implementation-ready tools? Bibliometric analysis—a quantitative approach to examining publication patterns, collaboration networks, and knowledge structures [30, 31]—provides an objective framework to address these questions. By systematically mapping the current state of knowledge, bibliometric methods can identify research trends, highlight underexplored areas, and inform strategic priorities for future investigation. Rather than evaluating the clinical effectiveness of individual AI systems, this review maps the scope and structure of the field—what is being studied, how, and where—to identify trends and research gaps.

## Methods

### Study design and objectives

This study employed a cross-sectional bibliometric design to systematically map the landscape of artificial intelligence applications in eating disorder research. The primary objectives were to characterise publication trends over time, describe the distribution of AI methodologies and clinical applications across the literature, identify geographical patterns of research activity, and assess the translational readiness of the AI systems described. This study followed the PRISMA extension for Scoping Reviews (PRISMA-ScR); the completed checklist is provided as Supplementary File 1. This review was not prospectively registered, and no separately published protocol exists; the a priori methods are described in full below.

### Search strategy and database

A systematic literature search was conducted across three databases—PubMed (MEDLINE), the Web of Science Core Collection, and Scopus—to ensure comprehensive and cross-validated coverage of the literature. The initial PubMed search was conducted on 20 November 2025 and subsequently cross-validated against Web of Science and Scopus. The search was restricted to articles published from 2016 onward. This starting point was selected because the modern wave of applied machine learning and deep learning in clinical research—including the techniques that dominate the present corpus—emerged from the mid-2010s; earlier work predates the methodological developments that define contemporary AI applications in eating disorder research.

The search strategy was conducted in two sequential steps for methodological transparency. The primary search targeted the most frequently studied and consistently indexed eating disorder diagnoses, namely anorexia nervosa, bulimia nervosa, and binge eating disorder. A secondary diagnostic-expansion search was then conducted to improve retrieval sensitivity for less-frequently indexed DSM-5 feeding and eating disorder categories, including avoidant/restrictive food intake disorder, pica, rumination disorder, and OSFED. The two searches were not treated as separate evidence pools; after within- and cross-database deduplication, all eligible records were combined into a single analytic corpus.

Two complementary Boolean search strings were applied to titles and abstracts. The primary search string targeted the three principal eating disorder diagnoses:

*("artificial intelligence" OR "machine learning" OR "deep learning" OR "neural network" OR "AI") AND ("eating disorder" OR "anorexia nervosa" OR "bulimia nervosa" OR "binge eating disorder").*

A supplementary search string was subsequently applied to ensure comprehensive coverage of additional DSM-5 feeding and eating disorder categories:

*("artificial intelligence" OR "machine learning" OR "deep learning" OR "neural network" OR "AI") AND ("ARFID" OR "avoidant restrictive food intake" OR "pica" OR "rumination disorder" OR "OSFED" OR "feeding disorder").*

The two-step strategy was adopted because the principal diagnoses (anorexia nervosa, bulimia nervosa, binge eating disorder) and the less-frequently indexed categories (ARFID, pica, rumination disorder, OSFED) are captured by distinct terminology; a sequential approach maximised retrieval sensitivity for the rarer diagnoses whilst keeping the primary search precise.

**Fig. 1:**
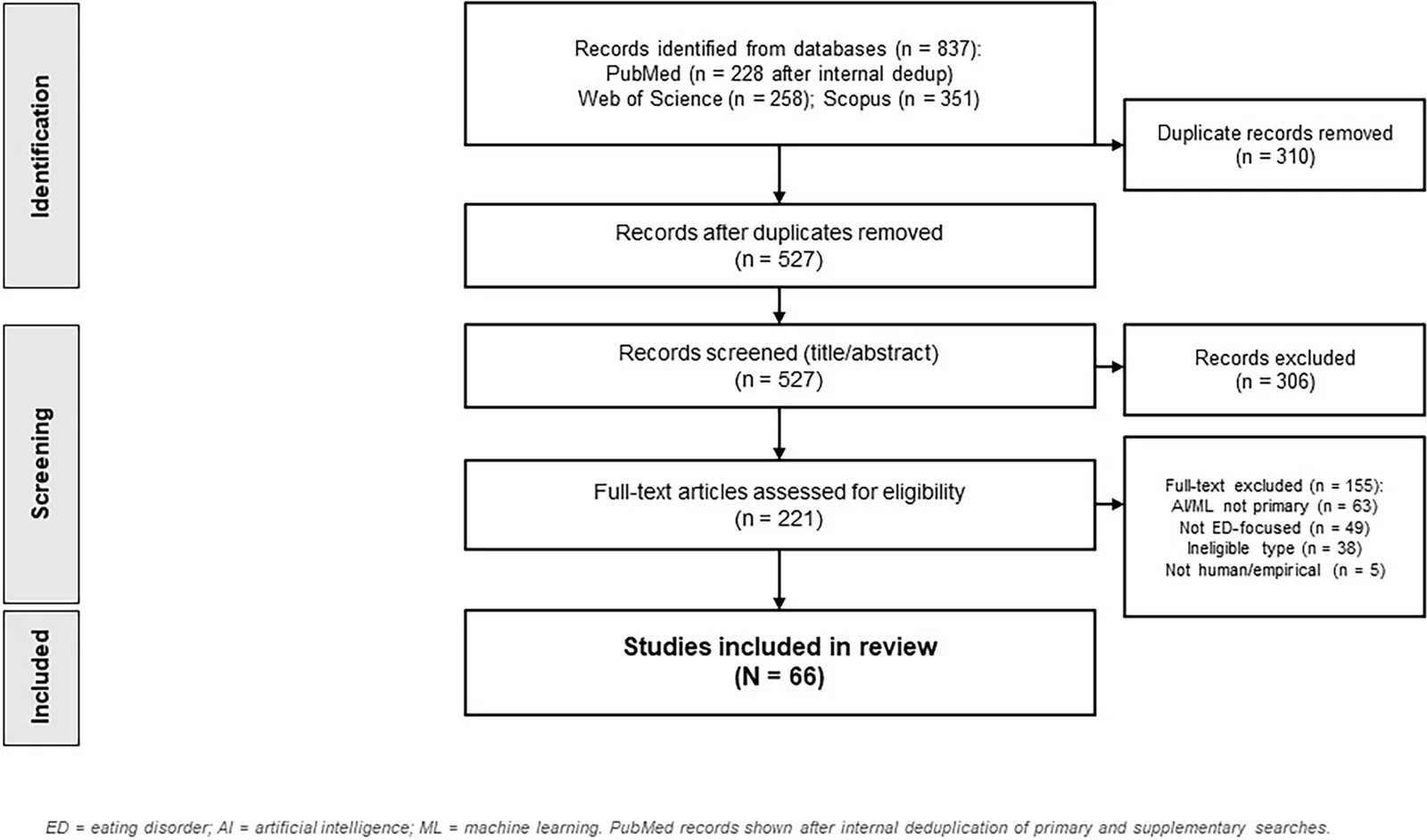
PRISMA 2020 flow diagram of study selection. ED eating disorder; AI artificial intelligence. Records were identified across three databases (PubMed, Web of Science, Scopus). PubMed records are shown after internal deduplication of the primary and supplementary searches

The PubMed primary search yielded 221 records and the supplementary search 165 records, which after deduplication comprised 228 unique PubMed records. Parallel searches in Web of Science (n = 258) and Scopus (n = 351) were then conducted using equivalent terms. Across all three databases, 837 records were identified (228 from PubMed, 258 from Web of Science, and 351 from Scopus). After removal of 310 duplicate records (within- and cross-database), 527 unique records underwent title and abstract screening, of which 306 were excluded. The remaining 221 full-text articles were assessed for eligibility, and 66 met all inclusion criteria. The selection process is illustrated in Fig. 1.

### Review framework

The review question was structured according to the PCC (Population, Concept, Context) framework recommended for scoping reviews: Population—individuals with, or at risk of, eating disorders (anorexia nervosa, bulimia nervosa, binge eating disorder, or broadly defined disordered eating); Concept—the application of artificial intelligence or machine learning methods as the primary analytical approach; Context—empirical research published between 2016 and 2025, across clinical, neuroimaging, social-media, and sensor-based settings.

### Eligibility criteria

Articles were included if they met all of the following criteria: (1) original empirical research design (reviews, editorials, letters, protocols, and commentaries were excluded); (2) use of at least one AI or machine learning method as the primary analytical approach; and (3) focus on an eating disorder population or eating disorder-related outcomes, encompassing anorexia nervosa, bulimia nervosa, binge eating disorder, or broadly defined disordered eating samples. Articles were excluded if AI was used solely as a screening or administrative tool without investigating an eating disorder outcome, if findings were based on animal or preclinical models without direct clinical applicability, or if the publication language was other than English.

### Data extraction and bibliometric indicators

For each included article, the following variables were extracted into a structured database: PubMed identifier (PMID) where available, title, authors, year of publication, journal, country of the corresponding author, eating disorder type studied (anorexia nervosa, bulimia nervosa, binge eating disorder, or mixed/general), target population (adults, adolescents, mixed, or not specified), sample size, and study design. Bibliometric indicators were retrieved from the Web of Science Core Collection and included total citation count, annual citation counts from 2016 to 2025, journal five-year impact factor, and the h-index of the first author. Each included article was cross-referenced against the Web of Science Core Collection using its Digital Object Identifier (DOI). Of the 66 included studies, 65 (98.5%) were successfully located in Web of Science; one article was absent from the Web of Science index and was therefore excluded from citation-based analyses. For the bibliographic coupling and co-citation analyses, which require full cited-reference lists, three articles lacked structured cited-reference records in the Web of Science Core Collection; for these, reference lists were extracted manually from the full texts and standardised to the same format. Although the supplementary search string targeted the less commonly indexed DSM-5 categories (ARFID, pica, rumination disorder, and OSFED), no eligible studies focussed primarily on these diagnoses, so no separate category was required and all included studies were classified as anorexia nervosa, bulimia nervosa, binge eating disorder, or mixed/general.

### Classification framework

Each study was classified along four predefined dimensions: AI methodology, clinical application domain, data source, and implementation readiness. Because no standardised taxonomy exists for AI applications in eating disorder research, category definitions were developed a priori and applied through an explicit coding protocol with defined category boundaries to minimise classification bias. Each dimension was treated as mutually exclusive at the study level; where a study could map to more than one category, assignment followed a predefined dominance rule specified for each dimension.

### AI methodology

AI methods were categorised into four mutually exclusive groups. The Machine Learning category encompasses studies employing traditional supervised and unsupervised learning algorithms, including support vector machines, random forest, logistic regression, decision trees, XGBoost, elastic net, and k-nearest neighbours. The Deep Learning category was reserved for studies in which a neural-network architecture (e.g. convolutional, recurrent, or long short-term memory networks; ResNeXt) constituted the primary methodological contribution, independent of the modality-defined NLP and Machine Learning categories (see the dominance rule below). The Natural Language Processing (NLP) category covers studies in which NLP techniques constituted the primary analytical method, typically involving analysis of text data from social media, clinical notes, or linguistic patterns; studies in this category were classified as NLP regardless of the underlying algorithm type—whether traditional machine learning or transformer-based—consistent with established conventions in psychiatric AI research. The Conversational AI category comprises studies employing rule-based or AI-powered chatbot systems as the primary intervention or analytical tool. We acknowledge that NLP and deep learning are not mutually exclusive at the architectural level, as many contemporary NLP systems employ deep learning architectures such as BERT. We therefore report this overlap transparently: a substantial proportion of the 13 studies classified as NLP employed transformer or other deep learning architectures. This allows methodological prevalence to be interpreted under either a task-based or an architecture-based convention. Deep learning was retained as a separate category in Table 4 because it was defined a priori in the coding protocol, and we report the framework exactly as it was applied rather than collapsing categories post hoc. Under the dominance rule, the primary data modality and analytical task took precedence over algorithmic architecture: neural-network and transformer-based models applied to text data were coded as NLP, whereas neural networks applied to structured, neuroimaging, or sensor data—typically alongside or in comparison with classical algorithms—were coded as Machine Learning. No study in the corpus met the Deep Learning criterion; the zero count in Table 4 therefore reflects the classification convention rather than the absence of deep learning architectures.

### Clinical application domain

The primary clinical purpose of each AI system was classified into one of five mutually exclusive categories addressing the question: what clinical function does this AI system serve in practice? The Diagnosis category includes AI systems designed to identify an eating disorder or perform differential diagnosis at the individual clinical level. Risk Prediction covers models predicting eating disorder onset, relapse, or risk in specific clinical scenarios such as post-bariatric surgery. Treatment Outcome encompasses models predicting treatment response, illness trajectory, or responsiveness to digital or clinical interventions. Symptom Monitoring refers to AI approaches that detect or track eating disorder behaviours—including bingeing, purging, and dietary restriction—through wearable sensors, continuous glucose monitors, or social media. A Detection/Screening category was applied to studies whose primary function was case detection or population-level screening. The Diagnosis and Detection/Screening categories were distinguished by their primary objective: Diagnosis referred to studies aiming to identify or differentiate an eating disorder at the individual clinical level, whereas Detection/Screening referred to studies whose primary aim was case-finding at the population or community level. Studies in which AI constituted the primary treatment and efficacy was evaluated through a randomised controlled trial were classified under Treatment Outcome Prediction. For studies reporting more than one clinical application, the dominant purpose served as the determining criterion. As data source and clinical application were coded as independent dimensions, studies sharing a data modality could differ in application: for example, amongst sensor-based studies, those primarily tracking ongoing behaviour were coded as Symptom Monitoring, whereas those predicting a future episode were coded as Risk Prediction.

### Data source

Data sources were classified into seven categories based on the primary modality employed. The Neuroimaging category includes studies relying on functional MRI, diffusion tensor imaging, or structural MRI. Clinical Data covers information collected through clinical interviews, psychometric scales, demographic and anthropometric measures, or randomised controlled trial datasets. The Wearable/sensor category covers data from actigraphy, smartwatches, or continuous glucose monitors, whilst the EMA/smartphone category covers real-time data gathered via ecological momentary assessment applications. The Genomic/EHR category includes genomic sequencing data and electronic health record–derived datasets. Social Media covers text and image data collected from online platforms including Twitter, Reddit, and eating disorder forums. The Survey category refers to self-report questionnaire datasets collected outside a structured clinical assessment. Where more than one data source was used within a single study, the primary modality was assigned to the most specific applicable category.

### Implementation readiness assessment

Each study was assigned to one of three implementation readiness levels to assess the translational maturity of the described AI systems. The Lab Only level designates studies in which the AI model was tested exclusively in a research setting with no external validation or clinical pilot; these studies are typically characterised by small sample sizes, single-centre designs, or the absence of an independent validation dataset. The Pilot level applies to studies in which the model was evaluated in a real-world or multi-centre setting, indicating that a proof-of-concept or feasibility stage had been completed, but large-scale clinical deployment had not yet been achieved. The Clinically Deployed level is reserved for studies in which AI system efficacy was confirmed through a randomised controlled trial or the system was actively used in clinical practice at the time of publication. This framework was developed a priori for the descriptive purposes of the present review and is not intended to replace established implementation-science models; it is presented as an exploratory descriptive gradient of translational maturity rather than a validated readiness instrument.

### Quality appraisal

Reporting quality of the included studies was appraised using the TRIPOD+AI statement [32] for prediction-model studies and CONSORT-AI [33] for randomised trials of AI-based interventions. Studies that did not develop or validate a deployable prediction model—mechanistic neuroimaging analyses in which machine learning served only as a robustness check, descriptive linguistic analyses, and unsupervised clustering studies—were classified as not appraisable. Each appraisable study was rated item-by-item as fully reported, partially reported, or not reported, based solely on explicitly reported information.

### Inter-rater reliability

Literature search, study screening, and data extraction were performed by the first author (Y.C.). All 66 included studies were then independently and blindly classified according to the predefined taxonomies by two authors (Y.C. and M.S.). Inter-rater agreement across the full corpus was substantial (Cohen's κ = 0.80), and all discrepancies were resolved through consensus discussion until full agreement was reached.

### Statistical analysis

Descriptive statistics were used to summarise all variables. Continuous variables, including citation counts, sample sizes, h-index values, and impact factors, are reported as medians with interquartile ranges, given the non-normal distributions expected in bibliometric data. Categorical variables, including AI method, clinical application domain, data source type, implementation readiness level, country of corresponding author, and eating disorder type, are reported as frequencies and percentages. Publication trends were examined by plotting annual article counts across the study period. Citation trajectories were analysed using year-by-year counts retrieved from the Web of Science Core Collection. All analyses were performed using Python (version 3.11) with the pandas, matplotlib, and NetworkX libraries. No inferential statistical testing was performed, consistent with the descriptive nature of bibliometric methodology. Co-authorship and keyword co-occurrence networks were constructed using the same platform. Keywords appearing in fewer than two articles were excluded; full counting was applied. Synonymous keyword variants were merged prior to analysis: spelling and hyphenation variants (e.g. ‘machine learning’/‘machine-learning’) and singular/plural forms (e.g. ‘eating disorder’/‘eating disorders’) were consolidated, as were the abbreviation ‘anorexia’ with ‘anorexia nervosa’. Conceptually distinct terms were kept separate (e.g. ‘artificial intelligence’ was not merged with ‘machine learning’, and ‘binge eating’ as a behaviour was distinguished from ‘binge eating disorder’ as a diagnosis). Authors with fewer than two publications were excluded from the co-authorship network. Network structure was characterised quantitatively using density, modularity (via greedy community detection), degree centrality, and mean clustering coefficient. The normalised total citation (NTC) for each article was calculated as its total citation count divided by the mean citation count of all corpus articles published in the same year. Two additional bibliometric networks were constructed to characterise the intellectual structure of the field. A bibliographic coupling network linked pairs of included studies that shared two or more cited references, and a co-citation network linked reference pairs that were cited together by three or more included studies. Both networks were built in Python using NetworkX, and community structure was identified using the Louvain modularity-maximisation algorithm; thematic labels were assigned to each resulting cluster by the authors based on the predominant content of its constituent studies. Cited-reference data were obtained from the Web of Science Core Collection, with manual extraction for the three articles (as described above).

## Results

**Fig. 2:**
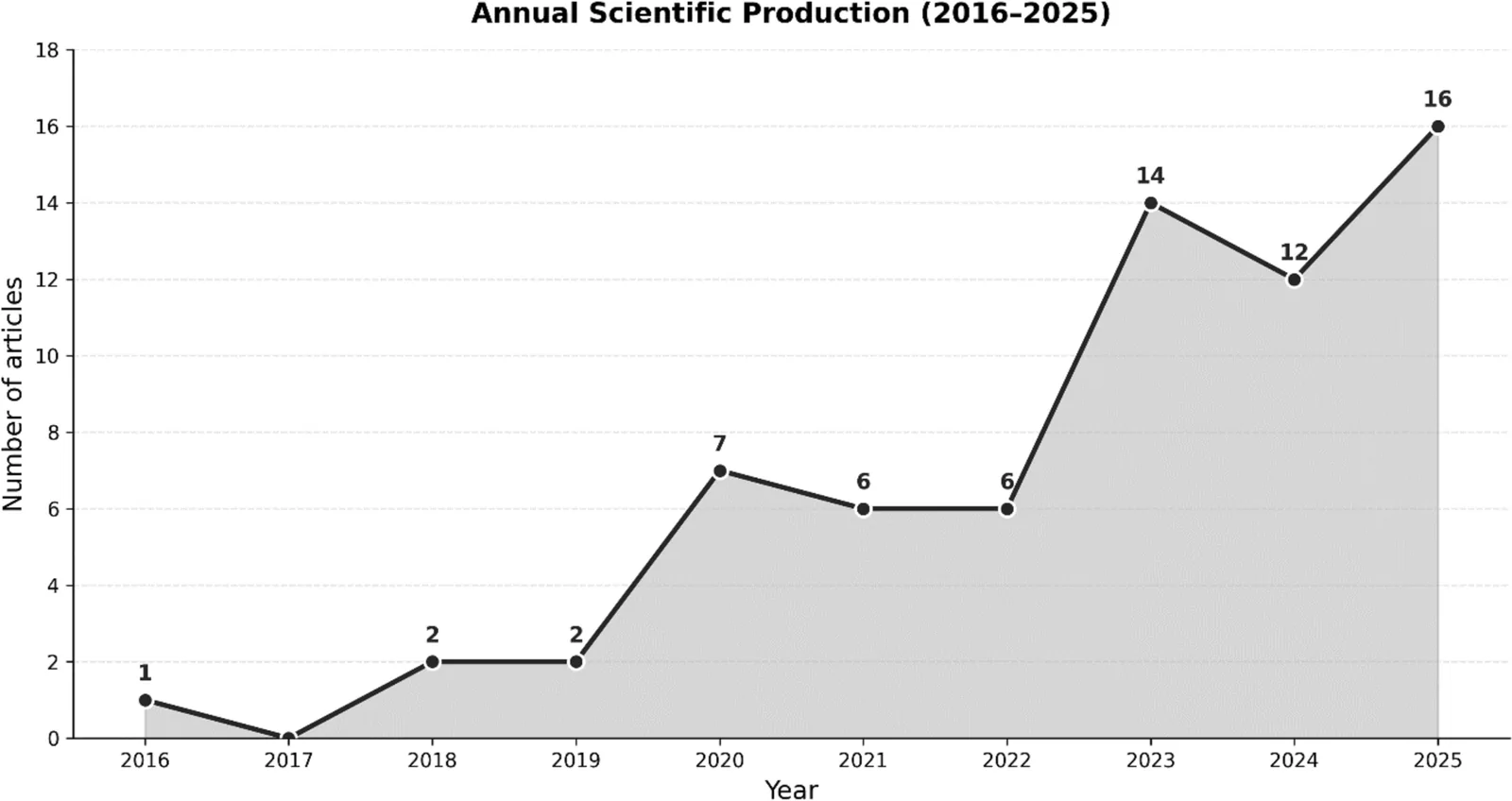
Annual Scientific Production (2016–2025). The shaded area represents the volume of publications per year (N = 66)

The final sample comprised 66 original empirical articles published between 2016 and 2025. Publication output rose from one article in 2016 to 16 in 2025 (Fig. 2). Output was lowest between 2016 and 2019 (range: 0–2 articles/year), increased to seven articles in 2020, was six in both 2021–2022, and rose to 14 in 2023, 12 in 2024, and 16 in 2025.

Fuller-Tyszkiewicz (n = 6) and Linardon (n = 5) were the most prolific contributors, followed by Spinczyk, Rojewska, and Greenwood (4 articles each) (Table 1). Publication output was distributed across 38 journals; the International Journal of Eating Disorders (n = 10, 15.2%) and Psychological Medicine (n = 8, 12.1%) were the most represented sources (Table 2).

**Table 1:** Most productive authors (≥ 4 articles)

Author	Articles (n)	Country
Fuller-Tyszkiewicz, Matthew	6	Australia
Linardon, Jake	5	Australia
Spinczyk, Dominik	4	Poland
Rojewska, Katarzyna	4	Poland
Greenwood, Christopher J	4	Australia

**Table 2:** Most productive journals in the corpus

Journal	Articles (n)	Percentage (%)
International Journal of Eating Disorders	10	15.2
Psychological Medicine	8	12.1
European Eating Disorders Review	4	6.1
JMIR Medical Informatics	3	4.5
Journal of Medical Internet Research	3	4.5
Applied Sciences	3	4.5
Frontiers in Psychiatry	2	3.0
Journal of Eating Disorders	2	3.0
Computer Methods and Programs in Biomedicine	2	3.0
Other (29 journals)	29	43.9

The ten most-cited articles are presented in Table 3. Lavagnino et al. (2018) and Haynos et al. (2021) recorded the highest total citation counts (50 and 49, respectively). Normalisation for publication year, however, altered this ranking: the highest normalised citation counts belonged to two natural language processing/social-media studies—Benítez-Andrades et al. (2022, NTC = 2.34) and Zhou et al. (2020, NTC = 2.15)—alongside Haynos et al. (2021, NTC = 2.07), rather than to the neuroimaging studies that led on raw counts.

**Table 3:** Ten most-cited articles in the corpus

First Author (Year)	Journal	Total Citations	Citations/Year	NTC
Lavagnino (2018)	Int J Eat Disord	50	6.25	1.27
Haynos (2021)	Psychol Med	49	9.80	2.07
Kyritsis (2021)	IEEE J Biomed Health Inform	44	8.80	1.86
Benítez-Andrades (2022)	JMIR Med Inform	32	8.00	2.34
Yan (2019)	Int J Eat Disord	31	4.43	1.75
Cyr (2018)	Hum Brain Mapp	29	3.62	0.73
Zhou (2020)	JMIR Med Inform	28	4.67	2.15
Forrest (2023)	Psychol Med	25	8.33	1.89
Burstein (2023)	Nat Genet	25	8.33	1.89
Espel-Huynh (2021)	Int J Eat Disord	21	4.20	0.89

*NTC* normalised total citations

**Fig. 3:**
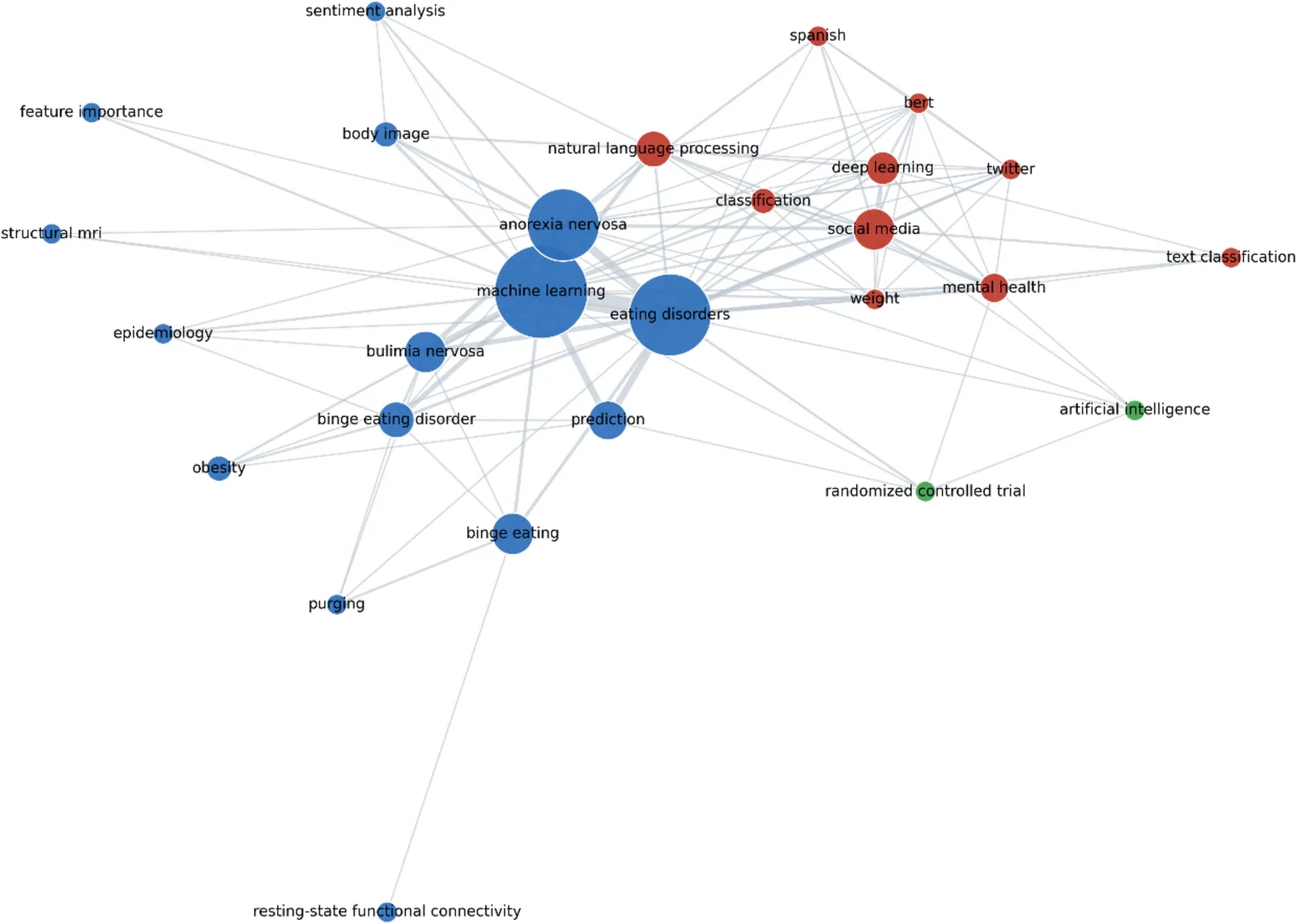
Keyword co-occurrence network of the 66 included studies. Nodes represent author-assigned keywords appearing in ≥2 articles (n = 27); node size reflects frequency and edge thickness reflects co-occurrence strength. Colours indicate three thematic clusters from modularity-based community detection. Synonymous variants were merged manually. Network metrics: density = 0.325, modularity = 0.205, mean clustering = 0.770, mean degree = 8.44

A total of 232 unique author-assigned keywords were identified. 'Machine learning' was the most frequent term (n = 40), followed by 'eating disorders' (n = 31) and 'anorexia nervosa' (n = 24). The keyword co-occurrence network (Fig. 3) comprised 27 keywords appearing in at least two articles (density = 0.325, modularity = 0.205) and identified three thematic clusters: a machine learning and clinical-prediction cluster (anorexia nervosa, bulimia nervosa, binge eating disorder, prediction), an NLP and social-media cluster (deep learning, Twitter, sentiment analysis), and a smaller cluster linking artificial intelligence with clinical trials.

**Fig. 4:**
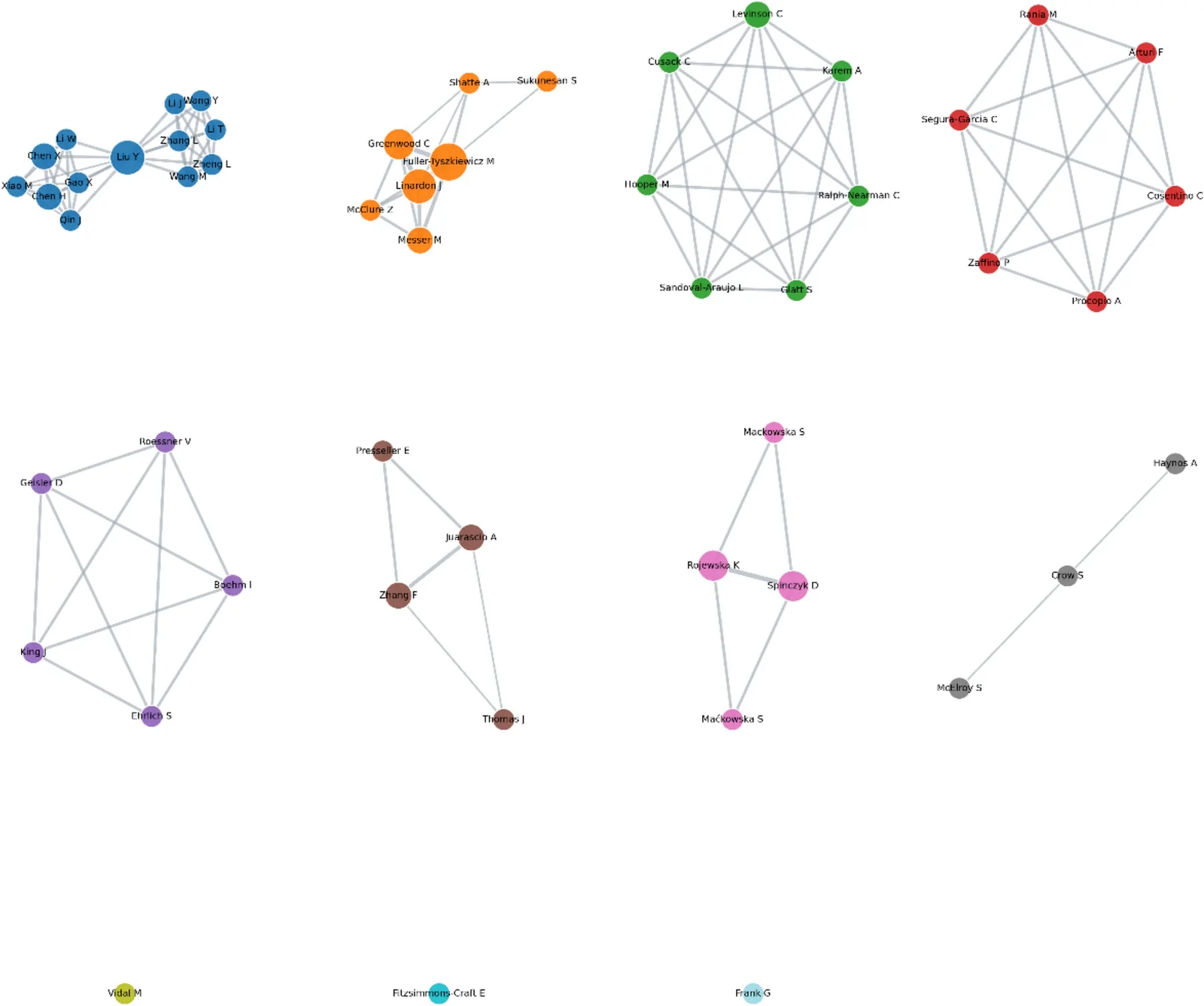
Co-authorship network of authors contributing to ≥2 of the 66 included studies (n = 52 authors). Node size reflects publication count; colour denotes separate collaboration components, arranged separately for readability. Network metrics: 52 nodes, 115 edges, density = 0.087, 11 disconnected components (largest = 13 authors), modularity = 0.804, mean clustering = 0.827

The co-authorship network comprised 52 authors with at least two publications, distributed across 11 disconnected components (density = 0.087; Fig. 4). The largest components corresponded to nationally based research groups, including teams in China, Australia (Fuller-Tyszkiewicz, Linardon, Greenwood), Italy, Germany, and Poland. High modularity (0.804) and low density reflect a fragmented collaboration structure with limited cross-group connectivity. Consistent with the relatively recent emergence of this research area, these findings indicate that author collaborations remain clustered within rather than between research groups.

Table 4 summarises the distribution of study characteristics. Machine learning was the dominant AI methodology (n = 52, 78.8%), followed by natural language processing (n = 13, 19.7%) and a single conversational-AI application (n = 1, 1.5%). No study was assigned to the pre-specified Deep Learning category (n = 0, 0.0%); under the task-based classification rule, studies using neural-network architectures were coded according to their primary analytical task (NLP or Machine Learning; see Methods and Table 4 footnote). The most common clinical applications were risk prediction (n = 22, 33.3%), diagnosis (n = 17, 25.8%), and treatment outcome prediction (n = 14, 21.2%). Clinical data were the most frequently used data source (n = 28, 42.4%), followed by neuroimaging (n = 12, 18.2%). The majority of studies remained at the laboratory stage (n = 51, 77.3%), with only two (3.0%) describing clinically deployed systems. Mixed/general ED samples (n = 24, 36.4%) and anorexia nervosa (n = 21, 31.8%) were the most studied populations, and adults constituted the primary target group (n = 39, 59.1%).

**Table 4:** Study characteristics (N = 66)

Category	Subcategory	n	%
AI Methodology	Machine Learning	52	78.8
	Natural Language Processing (NLP)	13	19.7
	Conversational AI	1	1.5
	Deep learning^a^	0	0.0
Clinical Application	Risk Prediction	22	33.3
	Diagnosis	17	25.8
	Treatment Outcome	14	21.2
	Detection/Screening	10	15.2
	Symptom Monitoring	3	4.5
Data Source	Clinical Data	28	42.4
	Neuroimaging	12	18.2
	Social media	10	15.2
	Survey	7	10.6
	Wearable/sensor	5	7.6
	EMA/smartphone	2	3.0
	Genomic/EHR	2	3.0
Implementation Readiness	Lab Only	51	77.3
	Pilot Study	13	19.7
	Clinically Deployed	2	3.0
Eating Disorder Type	Mixed / Multiple EDs	24	36.4
	Anorexia Nervosa (AN)	21	31.8
	Binge Eating Disorder (BED)	13	19.7
	Multiple specific diagnoses (AN+BN, BN+BED)	6	9.1
	Bulimia Nervosa (BN)	2	3.0
Target Population	Adults	39	59.1
	Mixed (Adults and Adolescents)	12	18.2
	Adolescents	8	12.1
	Not specified	7	10.6

^a^Pre-specified category retained for protocol fidelity. Classification followed a task-based dominance rule: studies employing neural-network architectures were assigned to NLP (text data) or Machine Learning (structured, neuroimaging, or sensor data) according to their primary analytical task, so no study was coded as Deep Learning at the primary level (see Methods, AI Methodology)

Across the corpus, the median sample size was 281 (IQR 70–728), the median journal five-year impact factor was 5.10 (IQR 3.80–6.70), the median Web of Science citation count was 7 (IQR 2–15), and the median first-author h-index was 11 (IQR 6–20) (Table 5).

**Table 5:** Bibliometric and descriptive indicators of the included studies (N = 66)

Indicator	Median	IQR	Range	n
Sample size	281	70–728	1–123,977	63
Journal five-year impact factor	5.10	3.80–6.70	0.6–37.4	65
Web of Science citation count	7	2–15	0–50	65
First-author h-index	11	6–20	1–57	65

Values are reported for studies with available data; sample size was not reported in 3 studies, and journal impact factor, citation count, and first-author h-index were unavailable for 1 study each

IQR interquartile range

**Fig. 5:**
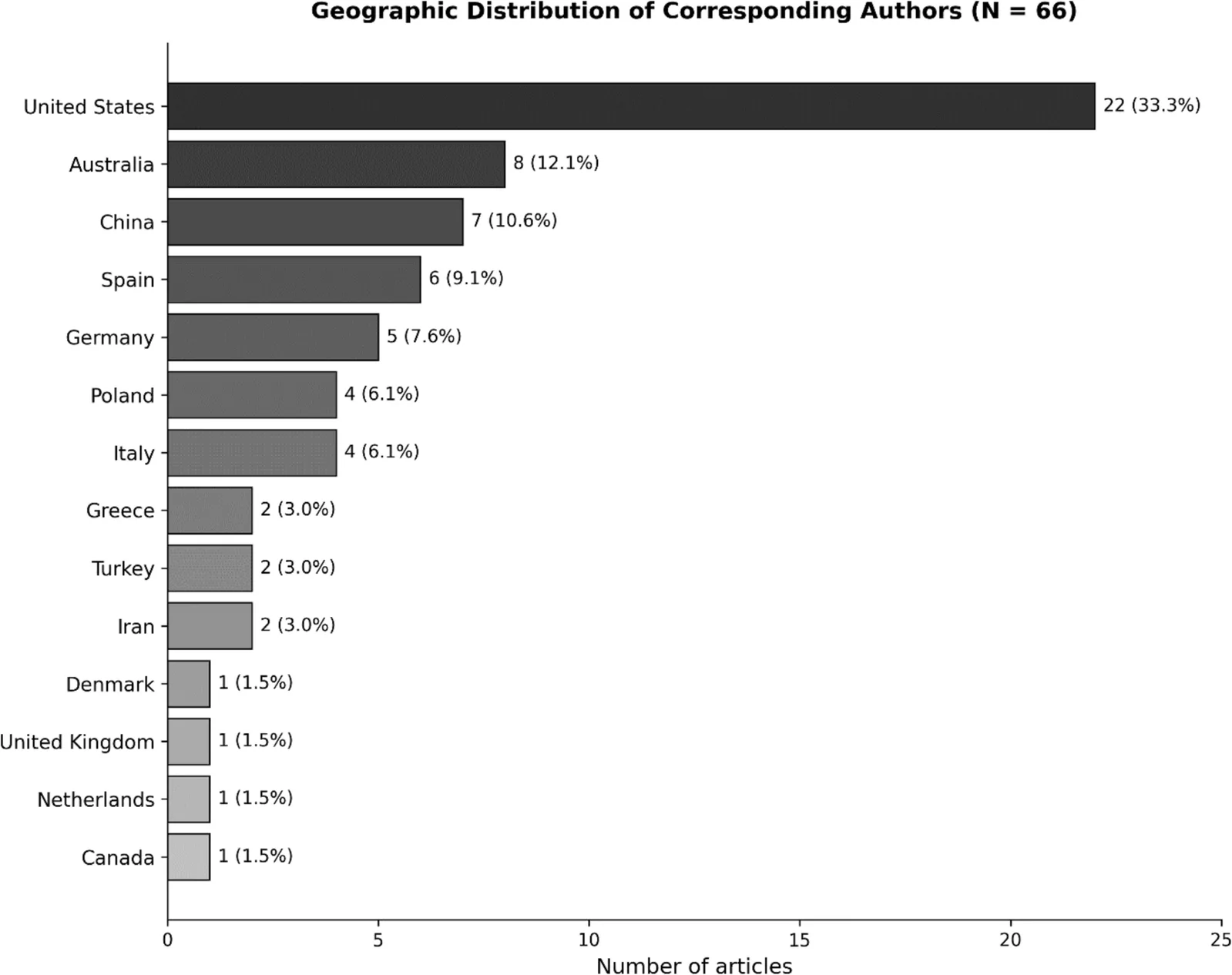
Geographical distribution of corresponding authors. Values represent the number of articles per country (N = 66)

Contributing authors were affiliated with institutions in 14 countries. The United States accounted for the largest share of publications (n = 22, 33.3%), followed by Australia (n = 8, 12.1%), China (n = 7, 10.6%), Spain (n = 6, 9.1%), and Germany (n = 5, 7.6%). No contributions were identified from Africa, South America, or Southeast Asia (Fig. 5).

Of the 66 included studies, 58 were appraisable with TRIPOD+AI, one (a chatbot randomised trial) with CONSORT-AI, and seven were classified as not appraisable (six neuroimaging or descriptive analyses in which machine learning was not a deployable model, and one unsupervised clustering study). Across all 58 TRIPOD+AI studies, a mean of 16.8 of 27 items were fully reported (item-level summary in Supplementary Table S1). Reporting was consistently strong for data source (100%) and analytical methods (100%), objectives (98%), interpretation versus prior work (98%), predictor definition (95%), clinical implications (97%), and study limitations (97%); most studies also reported internal validation (84%). In contrast, several items critical to clinical translation were almost universally absent: study registration was reported by none of the studies (0%), fairness or subgroup performance analysis by 2%, sample-size justification by 3%, full calibration assessment by 7%, and external validation on an independent dataset by 10%. Confidence intervals around performance estimates were fully reported by only 17%.

**Fig. 6:**
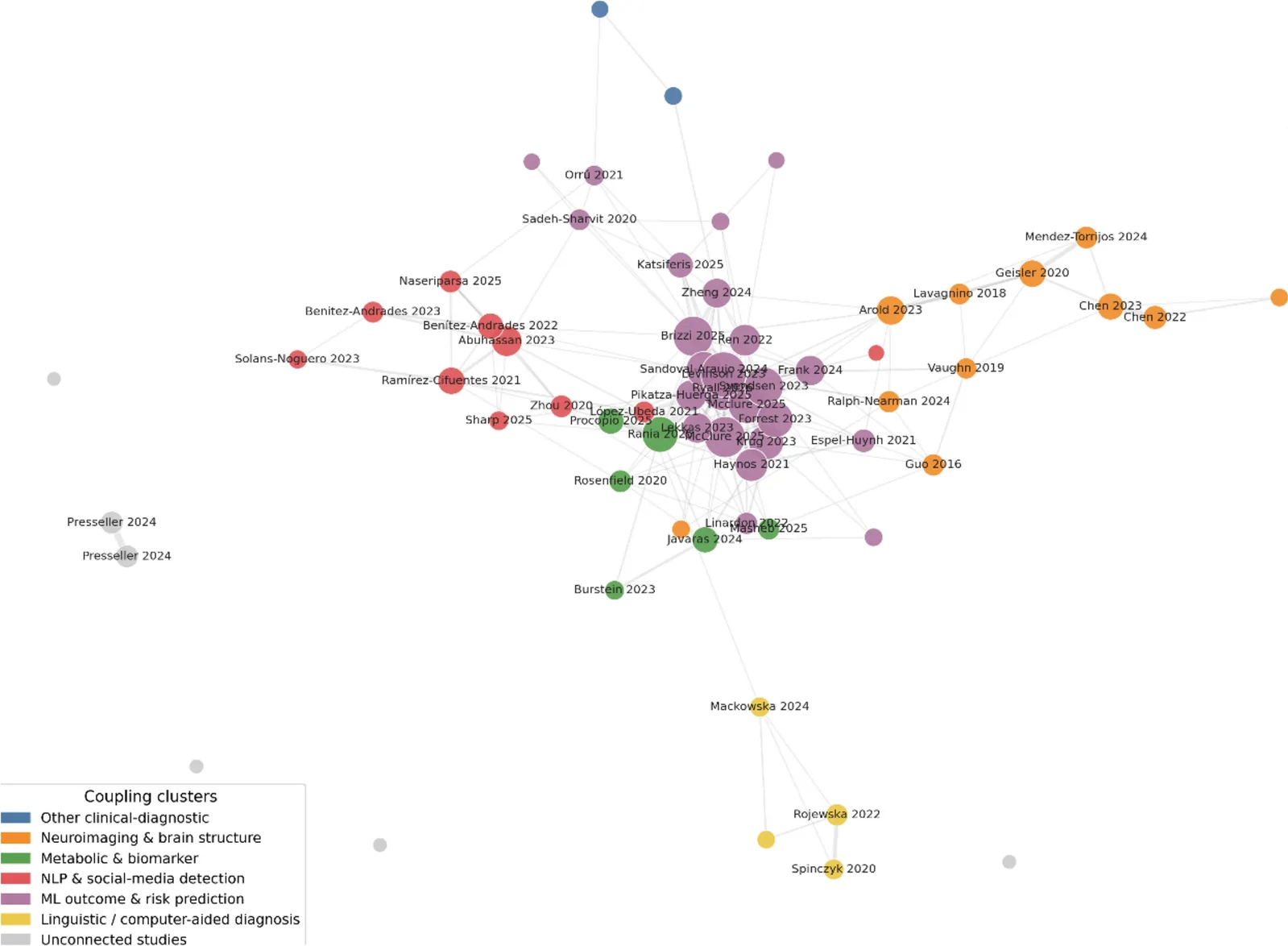
Bibliographic coupling network of the 66 included studies. Each node represents an included study; edges connect studies sharing at least two cited references, with edge thickness proportional to the number of shared references. Node size reflects coupling strength (weighted degree), and colours denote clusters identified by Louvain community detection. Network metrics: 66 nodes, 217 edges, density = 0.101, largest component = 57 studies, six clusters; seven studies showed no coupling links and appear as isolated nodes

**Fig. 7:**
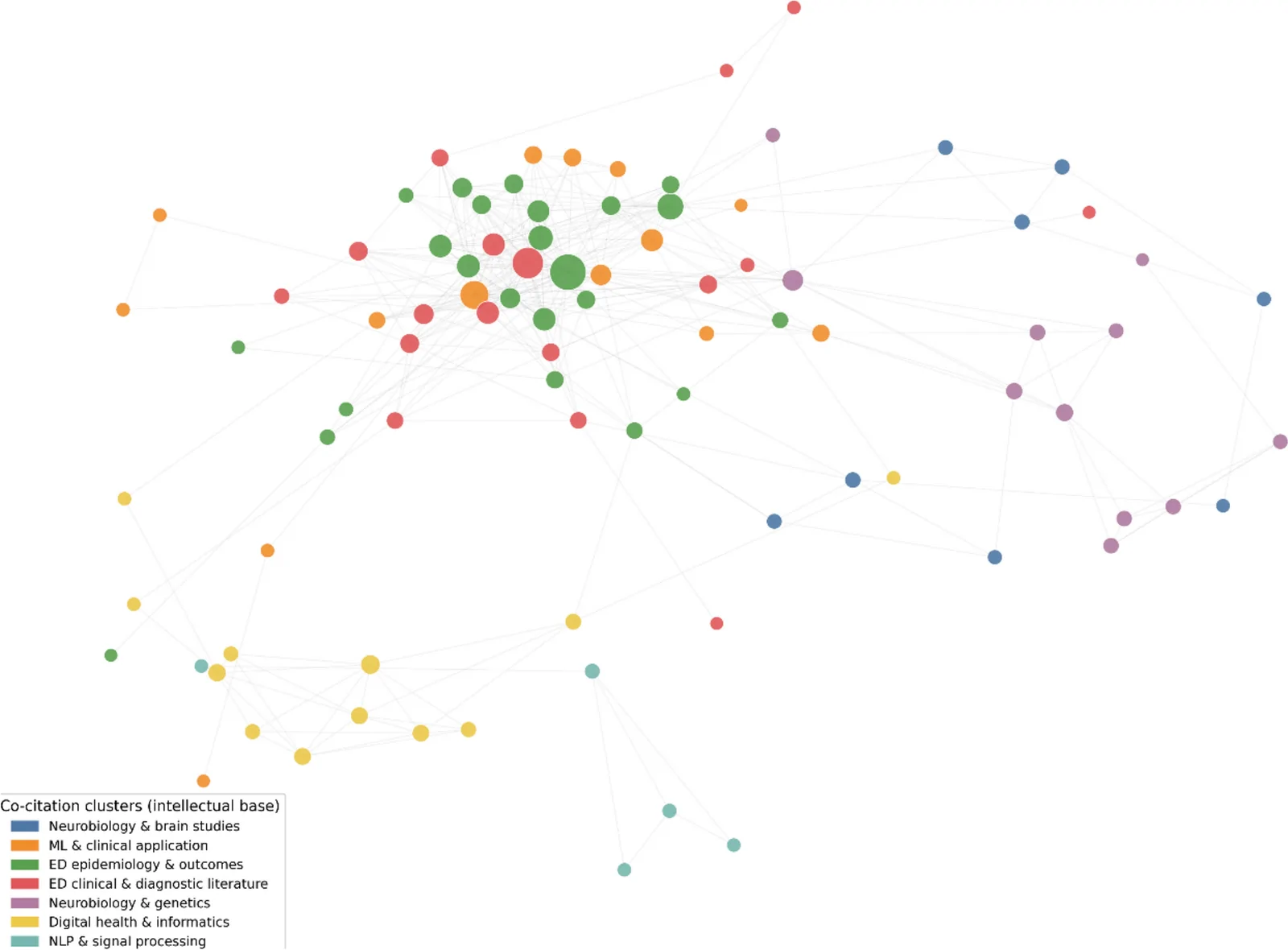
Co-citation network of references cited by the 66 included studies. Nodes represent references cited together by at least three included studies; edges represent co-citation frequency. Node size reflects co-citation count, and colours denote intellectual clusters from Louvain community detection. Network metrics: 90 nodes, 332 edges, seven clusters drawn from a pool of 3,422 unique cited references

Bibliographic coupling analysis, which links studies sharing common cited references, connected 59 of the 66 studies through shared references (217 coupling links; density = 0.101), with the largest single component comprising 57 studies and seven studies remaining unconnected. Modularity-based clustering identified six coupling communities that closely mirrored the thematic structure of the field: a machine learning outcome- and risk-prediction cluster, a neuroimaging cluster, a natural language processing and social-media cluster, a metabolic and biomarker cluster, a smaller nationally based linguistic/computer-aided-diagnosis cluster, and a small clinical-imaging cluster (Fig. 6). The co-citation network, mapping references frequently cited together across the corpus, comprised 90 frequently co-cited references (332 links) drawn from a pool of 3,422 unique cited references, organised into seven intellectual clusters spanning eating disorder epidemiology and outcomes, clinical and diagnostic literature, machine learning methodology, neurobiology and genetics, neuroimaging, digital health and informatics, and natural language processing (Fig. 7). The most frequently co-cited works clustered around foundational eating disorder epidemiology and outcome research together with core machine learning methodology, indicating that the field draws on an established clinical–epidemiological knowledge base whilst incorporating standard computational techniques.

## Discussion

This bibliometric analysis mapped 66 empirical studies published between 2016 and 2025, documenting the emergence of artificial intelligence research in eating disorder research as a recognisable field. The rapid growth in publication output—from a single article in 2016 to 16 in 2025—reflects a broader trend of expanding AI adoption across mental health research [10, 11] though the absolute volume of literature remains modest and the field is still in its early stages of development. The findings presented here provide a descriptive portrait of where the field currently stands: who is producing this research, what methods are being used, which clinical applications are prioritised, and how far these developments are from clinical implementation.

The strong dominance of traditional machine learning methods, which accounted for 78.8% of the corpus, is consistent with the broader psychiatric AI literature where supervised learning algorithms remain the primary analytical tool [10]. This reflects the availability of structured clinical datasets and the relative interpretability of methods such as random forests and support vector machines compared to deep learning architectures. The smaller representation of natural language processing (19.7%) and the single conversational AI application (1.5%) may partly reflect the relative novelty of these approaches; transformer-based NLP and large language models have only recently entered the clinical research agenda. Consistent with this trajectory, although NLP constituted a minority of the corpus, several NLP and social-media studies were amongst the highest-impact articles once citations were normalised for publication year, suggesting a subfield attracting growing attention even as traditional machine learning continues to dominate in volume. Deep learning architectures were present in the corpus but, under our task-based classification, were subsumed within the NLP and machine learning categories rather than counted separately. At the same time, the difficulty of accessing large and diverse datasets in eating disorder research remains a well-recognised barrier to the adoption of these methods [29]. Whether this distribution will shift in coming years—as larger datasets accumulate and transformer-based methods become more accessible to clinical researchers—remains to be seen, but the current picture suggests that the field is still in an early, largely exploratory phase of methodological development.

The distribution of clinical application domains reveals both the range of research activity and some notable imbalances. Risk prediction was the most studied application (33.3%), followed by diagnosis (25.8%) and treatment outcome prediction (21.2%). The relative emphasis on risk prediction and diagnosis is clinically relevant given that eating disorders are characterised by variable treatment response and high relapse rates [5]. Machine learning models that can identify individuals at risk of poor outcomes could theoretically support more personalised treatment allocation—an aspiration reflected in some of the most-cited articles in this corpus, including Haynos et al. [17], which demonstrated that machine learning outperformed traditional statistical approaches in predicting illness course and treatment response. However, these promising findings are not uncontested. Forrest et al. [34] found that machine learning models offered only minimal advantage over traditional statistical approaches in predicting BED treatment outcomes, and demonstrated that seemingly excellent model performance in some analyses was attributable to methodological inflation. The comparatively lower representation of detection/screening (15.2%) and symptom monitoring (4.5%) points to areas with considerable potential that have received less attention. These findings also draw attention to the gap between research activity and clinical implementation. The vast majority of studies (77.3%) remained at the laboratory stage, with only two describing clinically deployed systems. The Tessa chatbot incident—in which a conversational-AI system was deployed with inadequately tested generative features and produced harmful dietary content—illustrates the real-world consequences of moving towards clinical implementation without sufficient validation [23, 24]. Taken together, these observations suggest that the field is appropriately cautious, though the substantial gap between research output and actionable clinical tools remains a defining feature of the current landscape.

The predominance of clinical data (42.4%) and neuroimaging (18.2%) as data sources reflects longstanding traditions in eating disorder research, where clinical assessment and brain-based biomarker discovery have received sustained attention. Social media (15.2%) has emerged as a substantial third modality, whilst the relatively small contributions of ecological momentary assessment and wearable sensors (combined 10.6%), and survey data (10.6%) suggest that other passive and naturalistic data collection modalities—which offer particular promise for real-time symptom monitoring—are still emerging. These newer data sources introduce their own methodological and ethical challenges. Social media data raise concerns about consent, representation, and the potential for models to amplify harmful content [18], whilst the broader use of machine learning on population-level data has been shown to embed systematic biases that disadvantage already underrepresented groups [27]. Wearable and EMA data, meanwhile, present issues of ecological validity, participant burden, and the risk that continuous monitoring may itself affect disordered eating behaviours in vulnerable individuals.

Most studies focussed on adults (59.1%), whilst only 12.1% targeted adolescents exclusively. Given that eating disorder onset typically occurs during adolescence and young adulthood [35], this low proportion of adolescent-specific research is notable. Although a further 18.2% of studies included mixed adult and adolescent samples, research specifically designed around adolescent needs and developmental considerations remains limited. This gap is particularly concerning given that earlier intervention during adolescence is associated with significantly better long-term outcomes [35]; the absence of adolescent-focussed AI research therefore represents not merely a demographic imbalance but a missed clinical opportunity. Similarly, studies focussing exclusively on bulimia nervosa were rare (3.0%), suggesting that this disorder has received comparatively less targeted research attention relative to anorexia nervosa and binge eating disorder. This figure captures only studies focussing exclusively on bulimia nervosa; within this corpus, BN was more often visible in studies comparing it with anorexia nervosa than as the primary subject of investigation, and was rarely the principal focus of the predictive and treatment-oriented applications where AI research is most concentrated. This positions BN-specific prediction and treatment models as a comparatively underexplored area of AI research for this disorder. These gaps suggest that the current literature may not adequately reflect the populations most in need of improved diagnostic and therapeutic tools, and that findings from predominantly adult and anorexia nervosa samples may have limited generalisability to other groups.

The geographical concentration of research activity raises related but distinct concerns. The United States contributed 33.3% of included articles, with Australia, China, Spain, and Germany accounting for most of the remainder, and no contributions were identified from Africa, South America, or Southeast Asia. Eating disorder presentations, risk factors, and help-seeking behaviours are known to vary across cultural contexts [36, 37]; models trained exclusively on data from high-income Western populations may perform poorly when applied elsewhere [27]. Algorithmic bias is a well-documented risk in clinical AI more broadly, and there is little reason to assume that eating disorder models are exempt from these limitations. Broadening the geographical base of this research is therefore not simply a matter of representational fairness—it is a prerequisite for developing models that are clinically valid across diverse populations. Because geographical attribution was based on corresponding author affiliation, contributions from underrepresented regions embedded within multinational collaborations may not be fully captured; the apparent absence of output from Africa, South America, and Southeast Asia should therefore be interpreted with caution.

The fragmented co-authorship landscape documented here mirrors the geographical concentration of research activity. Collaborative ties remained confined within individual research groups, largely along national lines, with no cross-cluster connections identified. This pattern suggests that AI research in eating disorder research is developing in parallel silos rather than through the kind of sustained multidisciplinary exchange that translational progress requires. Establishing cross-institutional and international collaborations—particularly ones that bridge computational expertise with clinical knowledge—will be a necessary step towards developing models that are both methodologically robust and clinically applicable.

The bibliographic coupling and co-citation analyses add a further layer to this picture. The coupling structure showed that, despite the fragmented collaboration network, studies are thematically interconnected through shared references, clustering into coherent research fronts—machine learning prediction, neuroimaging, NLP/social-media, and metabolic research. The co-citation analysis indicated that these methodologically diverse subfields nonetheless rest on a shared intellectual foundation of eating disorder epidemiology and outcome research combined with standard machine learning methodology. Together, these findings suggest that, although the field is socially fragmented (distinct author groups), it is intellectually convergent (drawing on a common knowledge base)—a pattern consistent with an emerging field that has established shared theoretical foundations but has not yet developed dense cross-group collaboration.

The implementation readiness profile of this literature is sobering. The overwhelming majority of studies (77.3%) described models tested exclusively in research settings, with no external validation or clinical piloting. Only two studies described clinically deployed applications. This translational gap is not unique to eating disorders—it is a well-described challenge in clinical AI more broadly [29]—but it takes on particular significance in a field where vulnerable individuals may be exposed to inadequately tested interventions. The cautious attitudes observed amongst clinicians and people with lived experience in parallel survey research are therefore well-founded. Linardon and colleagues [9] found that whilst 59% of eating disorder clinicians were already using AI tools in their practice, most commonly ChatGPT for administrative tasks, fewer than one-third believed that AI would significantly improve client outcomes, and only a minority expressed enthusiasm for its broader integration. Both clinicians and patients widely agreed that AI is unlikely to replicate the relational and empathic dimensions of therapeutic care, even as they acknowledged its potential for administrative efficiency and between-session support. More recent qualitative work with eating disorder and AI professionals has similarly highlighted that successful implementation will require not only technically robust models but also clinician training, institutional infrastructure, and careful attention to patient safety and data governance [25].

A complementary perspective comes from a survey of eating disorder researchers conducted by the *International Journal of Eating Disorders* Executive Committee [38]. Nearly 70% of researchers in the field reported using generative AI tools to support their work, primarily for proofreading and coding tasks, with ChatGPT the most commonly used platform. Yet enthusiasm was tempered by recognition of real limitations: concerns about inaccurate outputs, risks to participant data confidentiality, and the potential for generative AI to reduce critical thinking or reinforce harmful stereotypes were widely endorsed. More than half of respondents anticipated that generative AI would increase research volume but reduce the depth of scholarly output—a concern that is directly relevant to the interpretation of bibliometric trends: the accelerating publication counts documented here may in part reflect AI-assisted writing and workflow efficiency rather than a proportional expansion of methodological rigour or clinical insights.

Looking ahead, several priorities emerge from the patterns documented here. Greater attention to adolescent populations and to eating disorder types beyond anorexia nervosa would better align research effort with clinical need. Expanding geographical representation—through international collaborations and investment in data infrastructure outside high-income Western settings—is a necessary condition for developing models that are equitable as well as accurate. The field would benefit from a shift in emphasis from model development to model validation: independent replication of promising findings, prospective clinical pilots, and ultimately randomised evaluations of AI-supported interventions. This shift will require not only technical progress but also the engagement of clinicians, patients, and regulatory bodies in shaping how AI is integrated into care. The low rate of clinical deployment documented here, combined with the caution expressed by clinicians and patients in survey research [9, 25], suggests that the field is not yet at risk of premature adoption, but also that substantial collaborative work remains to be done before the promising pilot results in this literature translate into real-world clinical benefit.

A structured reporting quality appraisal revealed a consistent pattern across the literature. Whilst studies reliably specified their algorithms, objectives, and internal validation procedures, the elements most essential for clinical deployment were rarely reported: none of the appraised studies documented prospective registration, and no more than about one in ten reported model calibration (7%) or external validation on an independent dataset (10%). Fairness analyses and sample-size justifications were almost entirely absent. This pattern indicates that, despite frequent claims of diagnostic or clinical potential, the field's evidence base remains methodologically exploratory. This concern is compounded by the modest and highly variable sample sizes across the corpus (median 281, IQR 70–728), since models developed on small samples and validated only internally are particularly vulnerable to overfitting and optimistic performance estimates—precisely the pattern of methodological inflation demonstrated in this corpus by Forrest et al. [34]. The near-absence of external validation and calibration in particular tempers prevailing implementation-readiness claims and defines clear priorities for future work: prospective registration, independent external validation, calibration reporting, and subgroup/fairness evaluation.

### Strengths and limits

The principal strength of this study is its systematic and reproducible bibliometric approach, which provides an objective, quantitative portrait of AI research in eating disorders across a ten-year period. The use of predefined classification taxonomies, applied consistently across all 66 included studies, enhances the reliability and comparability of findings. The incorporation of citation-based indicators, co-authorship and keyword co-occurrence mapping, and co-citation and bibliographic coupling analyses offers a multidimensional view of the field's intellectual structure that goes beyond simple publication counts.

Several limitations should be acknowledged. Although the search spanned three databases (PubMed, Web of Science, and Scopus), it may not capture the full range of computer science and engineering venues—such as conference proceedings indexed in IEEE Xplore or the ACM Digital Library—in which AI methods applied to eating disorders are also published. The restriction to English-language articles may have excluded relevant work from non-English-speaking research communities. Although all studies were independently double-coded with substantial inter-rater agreement, some classification decisions inevitably required judgement, particularly for studies that spanned more than one category. The use of Web of Science citation data introduces further variability as citation counts are sensitive to time since publication and journal indexing coverage. Finally, bibliometric methods describe the surface features of a literature—what is being published, where, and by whom—but cannot assess methodological quality or clinical validity; and although reporting completeness was appraised against the TRIPOD+AI and CONSORT-AI statements, no formal risk-of-bias assessment was undertaken. Although the bibliometric component incorporates co-citation and bibliographic coupling analyses in addition to keyword and co-authorship mapping, other advanced techniques—such as thematic evolution mapping and citation-burst detection—were not applied, as the relatively young corpus, with citations still accumulating, would render such time-dependent analyses unstable.

## Conclusion

This bibliometric analysis documents the emergence of artificial intelligence research in eating disorders as a recognisable but still nascent field. Publication output has grown rapidly, yet the literature remains geographically concentrated, methodologically dominated by traditional machine learning, and largely confined to laboratory settings. Adolescent populations, bulimia nervosa, and low- and middle-income country contexts are systematically underrepresented—gaps that limit the generalisability of current findings to the populations most in need. Closing the distance between research output and clinical implementation will require independent model validation, prospective evaluation, and sustained collaboration between computational researchers, clinicians, people with lived experience, and regulatory bodies. These conclusions are descriptive and should be read in the light of the limitations outlined above, particularly the reliance on reviewer judgement for classification and the absence of a formal risk-of-bias assessment. The present findings do not answer whether any given AI system works; they reveal where the field is investing its attention, and where that attention needs to shift.

### What is already known on this subject?

Artificial intelligence and machine learning methods have been increasingly applied across mental health research, with individual studies demonstrating promising results in eating disorder diagnosis, outcome prediction, and symptom monitoring. However, no comprehensive synthesis has previously mapped the scope, methodological distribution, or translational readiness of this emerging literature.

### What does this study add?

This study provides the first systematic bibliometric map of AI research in eating disorders across a ten-year period, identifying critical gaps in population coverage, geographical representation, and clinical implementation. The findings demonstrate that the field is growing rapidly but remains methodologically narrow and largely confined to laboratory settings, with substantial collaborative work required before research advances translate into clinically validated tools.

## Supplementary Information

Below is the link to the electronic supplementary material.

Supplementary file 1.

Supplementary file 2.

## Data Availability

The datasets analysed during the current study are available from the corresponding author on reasonable request.

## Declarations

### AI-assisted writing

AI-assisted tools were used solely for language editing to improve readability, grammar, and style. No AI tool was used to generate, analyse, or interpret any scientific content. The authors take full responsibility for the integrity and accuracy of all content and approved the final manuscript.
