# Supplementary material for: Artificial intelligence in eating disorder research: a scoping review with bibliometric analysis of trends, methodologies, and translational readiness (2016–2025)
Source: Eat Weight Disord. 2026 Sep 20;31(1):89. doi: 10.1007/s40519-026-01905-w (PMC13690902; doi:10.1007/s40519-026-01905-w)
Supplement: Supplementary file 1 [file 40519_2026_1905_MOESM1_ESM.docx]

**Supplementary File PRISMA-ScR Checklist**

*Preferred Reporting Items for Systematic reviews and Meta-Analyses extension for Scoping Reviews (PRISMA-ScR).*

**Manuscript:** Artificial Intelligence in Eating Disorder Research: A Scoping Review with Bibliometric Analysis of Trends, Methodologies, and Translational Readiness (2016–2025). *Checklist item wording follows Tricco AC, et al. PRISMA-ScR: A Scoping Review Extension. Ann Intern Med. 2018;169(7):467–473.*

| **Section** | **#** | **PRISMA-ScR Checklist Item** | **Reported on / Location in manuscript** |
| --- | --- | --- | --- |
| **TITLE** | | | |
| *Title* | **1** | Identify the report as a scoping review. | Title. “Artificial Intelligence in Eating Disorder Research: A Scoping Review with Bibliometric Analysis of Trends, Methodologies, and Translational Readiness (2016–2025).” |
| **ABSTRACT** | | | |
| *Structured summary* | **2** | Provide a structured summary that includes (as applicable) background, objectives, eligibility criteria, sources of evidence, charting methods, results, and conclusions that relate to the review questions and objectives. | Abstract (Purpose, Methods, Results, Conclusions). Structured abstract reports objectives, three databases, search date (20 November 2025), 2016–2025 range, 66 included studies, classification taxonomies, bibliometric indicators, key results, and conclusions. |
| **INTRODUCTION** | | | |
| *Rationale* | **3** | Describe the rationale for the review in the context of what is already known. Explain why the review questions/objectives lend themselves to a scoping review approach. | Introduction, paragraphs 1–4. Rationale for mapping a fragmented, rapidly growing field; explicit statement that the review maps scope and structure rather than evaluating clinical effectiveness. |
| *Objectives* | **4** | Provide an explicit statement of the questions and objectives being addressed with reference to their key elements (e.g., population or participants, concepts, and context) or other relevant key elements used to conceptualize the review questions and/or objectives. | Introduction (final paragraph) and Methods → Study Design and Objectives; review question framed with the PCC framework in Methods → Review Framework. |
| **METHODS** | | | |
| *Protocol and registration* | **5** | Indicate whether a review protocol exists; state if and where it can be accessed (e.g., a Web address); and if available, provide registration information, including the registration number. | Not registered. No published a priori protocol; the review was not prospectively registered. Stated in this checklist; a corresponding limitation acknowledgement can be added on request. |
| *Eligibility criteria* | **6** | Specify characteristics of the sources of evidence used as eligibility criteria (e.g., years considered, language, and publication status), and provide a rationale. | Methods → Eligibility Criteria (inclusion/exclusion). Years (2016–) with rationale in Methods → Search Strategy and Database; English-language restriction stated; empirical original-research requirement specified. |
| *Information sources* | **7** | Describe all information sources in the search (e.g., databases with dates of coverage and contact with authors to identify additional sources), as well as the date the most recent search was executed. | Methods → Search Strategy and Database. Three databases — PubMed (MEDLINE), Web of Science Core Collection, Scopus; search executed 20 November 2025. |
| *Search* | **8** | Present the full electronic search strategy for at least 1 database, including any limits used, such that it could be repeated. | Methods → Search Strategy and Database. Full Boolean primary and supplementary search strings reproduced verbatim; title/abstract fields and 2016– limit specified. |
| *Selection of sources of evidence* | **9** | State the process for selecting sources of evidence (i.e., screening and eligibility) included in the scoping review. | Methods → Search Strategy and Database (record counts, deduplication, title/abstract and full-text screening) and Figure 1 (PRISMA flow diagram); Methods → Interrater Reliability (screening performed by Y.C.). |
| *Data charting process* | **10** | Describe the methods of charting data from the included sources of evidence (e.g., calibrated forms or forms that have been tested by the team before their use, and whether data charting was done independently or in duplicate) and any processes for obtaining and confirming data from investigators. | Methods → Data Extraction and Bibliometric Indicators and Methods → Interrater Reliability. Structured extraction database; double, independent, blinded classification by two authors (Y.C., M.S.); Cohen’s κ = 0.80; disagreements resolved by consensus. |
| *Data items* | **11** | List and define all variables for which data were sought and any assumptions and simplifications made. | Methods → Data Extraction and Bibliometric Indicators (extracted variables and bibliometric indicators) and Methods → Classification Framework with its four sub-sections (AI Methodology, Clinical Application Domain, Data Source, Implementation Readiness), each with explicit category definitions and dominance rules. |
| *Critical appraisal of individual sources of evidence* | **12** | If done, provide a rationale for conducting a critical appraisal of included sources of evidence; describe the methods used and how this information was used in any data synthesis (if appropriate). | Methods → Quality Appraisal. Reporting quality appraised with TRIPOD+AI (prediction-model studies) and CONSORT-AI (AI-intervention trials); item-by-item rating; non-appraisable study types defined. Results and Supplementary Table S1 report item-level completeness; Discussion integrates the appraisal. |
| *Synthesis of results* | **13** | Describe the methods of handling and summarizing the data that were charted. | Methods → Statistical Analysis. Descriptive frequencies/percentages and medians with IQR; NTC definition; keyword co-occurrence, co-authorship, bibliographic coupling, and co-citation network construction (Python/NetworkX, Louvain/greedy community detection); no inferential testing. |
| **RESULTS** | | | |
| *Selection of sources of evidence* | **14** | Give numbers of sources of evidence screened, assessed for eligibility, and included in the review, with reasons for exclusions at each stage, ideally using a flow diagram. | Results (opening) and Methods → Search Strategy and Database; Figure 1 (PRISMA-ScR flow diagram): 837 identified → 527 screened → 221 full-text → 66 included. |
| *Characteristics of sources of evidence* | **15** | For each source of evidence, present the charted data on which data were charted and provide the citations. | Results and Tables 1–5; Supplementary Appendix (per-study appraisal, N = 66). Study-level characteristics (authors, journals, most-cited articles, descriptive indicators) tabulated. |
| *Critical appraisal within sources of evidence* | **16** | If done, present data on critical appraisal of included sources of evidence (see item 12). | Results (TRIPOD+AI item-level completeness paragraph); Supplementary Table S1 (item-level, n = 58) and Supplementary Appendix (per-study scores). |
| *Results of individual sources of evidence* | **17** | For each included source of evidence, present the relevant data that were charted that relate to the review questions and objectives. | Results and Tables 1–5; Figures 2–7 (annual production, keyword network, co-authorship, geographic distribution, bibliographic coupling, co-citation). Per-study appraisal in Supplementary Appendix. |
| *Synthesis of results* | **18** | Summarize and/or present the charting results as they relate to the review questions and objectives. | Results (all subsections): publication trends, AI methodology, clinical application, data source, implementation readiness, ED type, target population, geography, and network analyses summarised in Table 4 and the network figures. |
| **DISCUSSION** | | | |
| *Summary of evidence* | **19** | Summarize the main results (including an overview of concepts, themes, and types of evidence available), link to the review questions and objectives, and consider the relevance to key groups. | Discussion, paragraphs 1–10. Main findings synthesised and linked to the objectives; relevance to clinicians, patients, adolescents, under-represented diagnoses/regions, and policy discussed. |
| *Limitations* | **20** | Discuss the limitations of the scoping review process. | Discussion → Strengths and limits. Database/venue coverage, English-language restriction, coding judgement, citation-data variability, absence of formal risk-of-bias assessment, and non-application of thematic-evolution / citation-burst analyses discussed. |
| *Conclusions* | **21** | Provide a general interpretation of the results with respect to the review questions and objectives, as well as potential implications and/or next steps. | Discussion (closing) and Conclusion; “What is already known” / “What this study adds.” Implications and priorities (external validation, prospective evaluation, broader collaboration) stated. |
| **FUNDING** | | | |
| *Funding* | **22** | Describe sources of funding for the included sources of evidence, as well as sources of funding for the scoping review. Describe the role of the funders of the scoping review. | Declarations → Funding. “The authors did not receive support from any organization for the submitted work.” No funder role. |

*Note. Where indicated as “Not applicable,” the JBI/PRISMA-ScR guidance treats the item as optional for the review type (e.g., item 12 critical appraisal is optional in scoping reviews; here it was performed and reported). Item 5: this review was not prospectively registered and no separately published protocol exists; the a priori methods (taxonomies, eligibility, and analysis plan) are described in full within the Methods.*
