# Supplementary material for: Artificial intelligence in eating disorder research: a scoping review with bibliometric analysis of trends, methodologies, and translational readiness (2016–2025)
Source: Eat Weight Disord. 2026 Sep 20;31(1):89. doi: 10.1007/s40519-026-01905-w (PMC13690902; doi:10.1007/s40519-026-01905-w)
Supplement: Supplementary file 2 [file 40519_2026_1905_MOESM2_ESM.docx]

**Supplementary Table S1. TRIPOD+AI Item-Level Reporting Completeness (n = 58 prediction-model studies)**

Number of studies rated Y (fully reported), P (partially reported), N (not reported), or NA (not applicable) for each TRIPOD+AI item, with the percentage fully reporting each item. Values are pooled across all 58 TRIPOD+AI studies from a single item-level appraisal matrix.*.*

| **Item #** | **TRIPOD+AI Item** | **Fully (Y)** | **Partial (P)** | **Not (N)** | **NA** | **% Fully Reported** |
| --- | --- | --- | --- | --- | --- | --- |
| 1 | Title identifies prediction model | 36 | 20 | 2 | 0 | **62%** |
| 2 | Structured abstract | 45 | 13 | 0 | 0 | **78%** |
| 3 | Background & existing models | 55 | 3 | 0 | 0 | **95%** |
| 4 | Objectives | 57 | 1 | 0 | 0 | **98%** |
| 5 | Data source & design | 58 | 0 | 0 | 0 | **100%** |
| 6 | Participants: setting/eligibility/dates | 29 | 29 | 0 | 0 | **50%** |
| 7 | Outcome definition | 54 | 4 | 0 | 0 | **93%** |
| 8 | Predictors/features | 55 | 3 | 0 | 0 | **95%** |
| 9 | Sample-size justification | 2 | 10 | 45 | 1 | **3%** |
| 10 | Missing-data handling | 23 | 18 | 16 | 1 | **40%** |
| 11 | Data preprocessing | 52 | 5 | 1 | 0 | **90%** |
| 12 | Analytical method/algorithm | 58 | 0 | 0 | 0 | **100%** |
| 13 | Class-imbalance handling | 20 | 8 | 22 | 8 | **34%** |
| 14 | Model output | 57 | 1 | 0 | 0 | **98%** |
| 15 | Training/tuning/selection | 36 | 21 | 1 | 0 | **62%** |
| 16 | Internal validation | 49 | 8 | 1 | 0 | **84%** |
| 17 | Discrimination and calibration | 4 | 53 | 1 | 0 | **7%** |
| 18 | External validation | 6 | 2 | 50 | 0 | **10%** |
| 19 | Participant flow/characteristics | 50 | 6 | 1 | 1 | **86%** |
| 20 | Performance with confidence intervals | 10 | 25 | 23 | 0 | **17%** |
| 21 | Model presentation | 36 | 21 | 1 | 0 | **62%** |
| 22 | Fairness/subgroup analysis | 1 | 23 | 32 | 2 | **2%** |
| 23 | Limitations | 56 | 2 | 0 | 0 | **97%** |
| 24 | Interpretation vs prior work | 57 | 1 | 0 | 0 | **98%** |
| 25 | Clinical implications | 56 | 2 | 0 | 0 | **97%** |
| 26 | Study registration | 0 | 1 | 57 | 0 | **0%** |
| 27 | Open science (code/data) | 11 | 33 | 14 | 0 | **19%** |

Mean items fully reported: 16.8/27 across the 58 TRIPOD+AI studies. This item-level summary reflects the 58 TRIPOD+AI studies and excludes the 1 CONSORT-AI study (Sharp et al., appraised against its own 17-item checklist) and the 7 not-appraisable studies. The most consistently under-reported items were study registration (0%), fairness/subgroup analysis (2%), sample-size justification (3%), calibration (7%), and external validation (10%).
